# Association between Sleep Duration and Perceived Stress: Salaried Worker in Circumstances of High Workload

**DOI:** 10.3390/ijerph15040796

**Published:** 2018-04-19

**Authors:** Dong-Woo Choi, Sung-Youn Chun, Sang Ah Lee, Kyu-Tae Han, Eun-Cheol Park

**Affiliations:** 1 Department of Public Health, Graduate School, Yonsei University, 03772 Seoul, Korea; cdw6027@yuhs.ac (D.-W.C.); csy6909@yuhs.ac (S.-Y.C.); ivory0817@yuhs.ac (S.A.L.); 2 Institute of Health Services Research, Yonsei University, 03772 Seoul, Korea; 3 Department of Policy Research Affairs, National Health Insurance Service Ilsan Hospital, 10444 Koyang, Korea; kthan.phd@gmail.com; 4 Department of Preventive Medicine, Yonsei University College of Medicine, 03772 Seoul, Korea

**Keywords:** sleep duration, stress awareness, salaried workers

## Abstract

The aim of this study was to find the association between sleep duration and perceived stress in salaried workers according to occupational categories and which lifestyle factors affected those correlations in South Korea. This study used data from the 2015 Community Health Survey (CHS). The self-reported sleep duration was used as the dependent variable in this study. We explored sleep duration and stress awareness among salaried workers, as well as household income and educational level with multiple logistic regression analysis. Salaried workers who slept for five or less hours had a higher odds ratio for high-stress awareness (OR: 1.86, 95% CI: 1.74–1.98). Stress awareness is associated with short sleep duration; specialized workers, office workers, those with above mid-high household income and graduate, university, or college level workers especially need to sleep adequately to manage stress.

## 1. Introduction

From childhood to adulthood, everyone lives under stress. Stress has been known as major risk factor for serious illnesses such as depression, suicide, hypertension, and all-cause mortality [1,2]. Therefore, numerous studies focus on the need for stress management to improve the health of individuals. However, stress management is not easy for salaried workers who work for five or more days per week, especially in South Korea. South Korea has the second of the longest working hours in the world. South Korean salaried workers spend about 2069 h at work annually, which is 305 h more than Organisation for Economic Co-operation and Development (OECD) countries in 2015–2016 [3]. In particular, excessive workload and business hours are becoming a serious problem in South Korea. Moreover, frequent overwork time and drinking culture in the workplace until late at night make workers more exhausted; consequently, it might also affect leisure time and sleep time, and cause stress [4,5]. 

On the other side, the average sleep duration in South Korea is 7.7 h, which is less than OECD countries average sleeping length of 8.3 h [6]. Sleep duration is also a very important factor. In modern society, many people could not sleep in order to accommodate changes in their daily schedules or to prioritize other activities [7]. Appropriate sleeping has been known as a factor to increase a quality of life as well as a mental health. Previous studies have reported that the recommended duration of sleep for adults is 7 to 9 h per day [8,9]. It has come to represent a lifestyle factor which is able to be modified in much the same way as other health-related behaviors [7]. There is evidence to suggest that insufficient sleep may have a negative impact on mood and affect, as well as have adverse effects on cardiovascular and immune function [10,11]. Many studies have revealed the association between sleep duration and stress; however, most of these studies focused on the effect of stress on sleeping time [12,13,14,15]. Therefore, it might not be enough to clearly assess whether sleep duration affects the incidence of stress among salaried workers in the workplace.

The aim of this study was to assess the association between sleep duration and stress awareness in salaried workers according to occupational categories and the lifestyle factors which affect those correlations.

## 2. Methods

### 2.1. Study Population

This study used data from the 2015 Community Health Survey (CHS) which was conducted by the Korea Centers for Disease Control and Prevention. Among 228,558 participants enrolled in this analysis, 86,007 participants were salaried employees. We excluded 18,582 participants who did not respond to questions about covariates, including stress awareness, sleep duration, sex, age, educational level, household income, occupational category, marital status, physical activity, alcohol consumption, smoking status, body mass index (BMI), perceived health status, depressed mood, and chronic disease. Accordingly, 67,425 participants were included in this study.

### 2.2. Variables

Self-reported sleep duration was used as the dependent variable in this study. In the survey questionnaires, participants were asked, “how many hours of sleep do you usually get at night?” They were divided into five categories according to their sleep duration (5 h or less, 6 h, 7 h, 8 h, and 9 h or more).

Self-rated stress awareness was the primary focus of this study. The questionnaire asked the question: “how much stressed do you feel in your daily life?” and participants answered: “I feel stress very much”, “I feel stress much”, “I feel stress little”, “I do not feel stress”. We divided the participants into two categories–the high stress group and low stress group–depending on their answers.

We used covariates, including sex, age (20 to 29, 30 to 39, 40 to 49, 50 to 59, and 60 or over), educational level (under high school, university or college, and graduate school), household income (low, mid-low, mid-high, and high), occupational category (specialized work, office work, service and sales work, agriculture, forestry, fishery, and machinery and simple labor), marital status (married-cohabiting, married-divorced, bereaved, separated, and single), physical activity (high, moderate, low), alcohol consumption (yes, no), smoking status (current smoker, ex-smoker, non-smoker), BMI (normal and underweight, overweight, obesity), perceived health status (health, normal, unhealthy), depressed mood (yes, no), and chronic disease (yes, no). Physical activity categories were divided based on metabolic equivalent tasks using the International Physical Activity Questionnaire-Short Form. BMI categories were based on their weight and height (BMI < 23 kg/m^2^: normal and underweight, BMI < 23~25 kg/m^2^: overweight, and BMI ≥ 25 kg/m^2^: obesity).

### 2.3. Statistical Analysis

For all analyses, we used the sampling weights variable provided by the CHS. We calculated the frequency and percentage of each categorized variable and performed chi-square test to identify significant differences among groups. Next, we performed multiple logistic regression to analyze the adjusted odds ratio (OR) of the self-reported stress awareness controlled for age, educational level, household income, occupational categories, marital status, physical activity, alcohol consumption, smoking status, BMI, perceived health status, depressed mood, and chronic disease. In the subgroup analysis, we performed multiple logistic regression by using stratified occupational categories, household income, and educational level with multiple logistic regression analysis. All statistical analyses were conducted with SAS version 9.4 (SAS Institute Inc., Cary, NC, USA). A *p*-value of less than 0.05 was considered statistically significant.

## 3. Results

The characteristics of the study population are shown in Table 1. About 39% of the participants slept for <5 h, about 30% and 25% of those who slept for 6 h and 7 h, respectively, 23% of those who slept for 8 h, and 24% of those who slept for 9+ h felt high stress. Twenty-eight percent of the participants of both sexes were stressed, and the number of participants who were highly stressed in young group was more than the older groups (20–29 years: 37%, 30–39 years: 35%, 40–49 years: 29%, 50–59 years: 22%, and older than 60 years: 16%). In the distribution according to job type, 33% of the office workers were found to get highly stressed, followed by specialized work (31%), services and promotion (30%), machinery and simple labor (23%), and agriculture, fishery and forestry (22%). Smokers (35%) reported a higher incidence of stress compared to non-smokers (23%) and ex-smokers (27%). According to perceived health status, participants who felt healthy had a lower level of stress awareness than those who felt normal or unhealthy (healthy: 23%, normal: 32%, and unhealthy: 45%). 

Table 2 shows the ORs for stress awareness for each variable. Regarding sleep duration, participants who slept for five or less hours had a higher OR for high stress awareness (OR: 1.86, 95% confidence interval [CI]: 1.74–1.98). Moreover, the odds ratios for high stress awareness according to increasing sleep duration were 1.27 (95% CI: 1.20–1.33) and 0.91 (95% CI: 0.84–0.98). However, groups that slept for at least 9 h did not show significantly higher levels of high stress awareness (OR: 0.92, 95% CI: 0.76–1.10). Compared to women, men had lower odds ratio for high stress awareness (OR: 0.86, 95% CI: 0.81–0.92). The ORs for high stress awareness for each 10-year age group were 3.44 (95% CI: 3.01–3.90), 2.93 (95% CI: 2.64–3.26), 2.16 (95% CI: 1.95–2.38), and 1.43 (95% CI: 1.29–1.59) for those in their 20s, 30s, 40s, and 50s compared with those over 60 years old, respectively. According to educational level, subjects who graduated from high school had lower odds ratios than those who graduated from college or higher (OR: 0.86, 95% CI: 0.78–0.96). The low household income group had a higher odds ratio compared to the high household income group (OR: 1.14, 95% CI: 1.05–1.23). However, the mid-low and mid-high household income groups were not significantly different from the high income group with regards to stress awareness. According to occupational category, specialized work, office work, service and sales work each had odds ratios of 1.35 (95% CI: 1.25–1.45), 1.43 (95% CI: 1.39–1.58), and 1.32 (95% CI: 1.22–1.40), respectively, compared to machinery and simple labor. Those who worked in agriculture, forestry, and fishery did not show significant levels of stress awareness compared to the machinery and simple labor group. Smokers had a 1.64 odds ratio (95% CI: 1.53–1.75) and ex-smokers had 1.10 odds ratio (95% CI: 1.02–1.18) for high stress awareness compared to non-smokers. Subjects who felt healthy had lower odds ratio (OR: 0.59, 95% CI: 0.56–0.62) and those who felt unhealthy had an odds ratio which was twice as high (OR: 2.06, 95% CI: 1.90–2.24) as those who felt normal. The participants who felt depressed had an odds ratio four times higher than those who did not feel depressed (OR: 3.92, 95% CI: 3.57–4.31).

**Figure 1:**
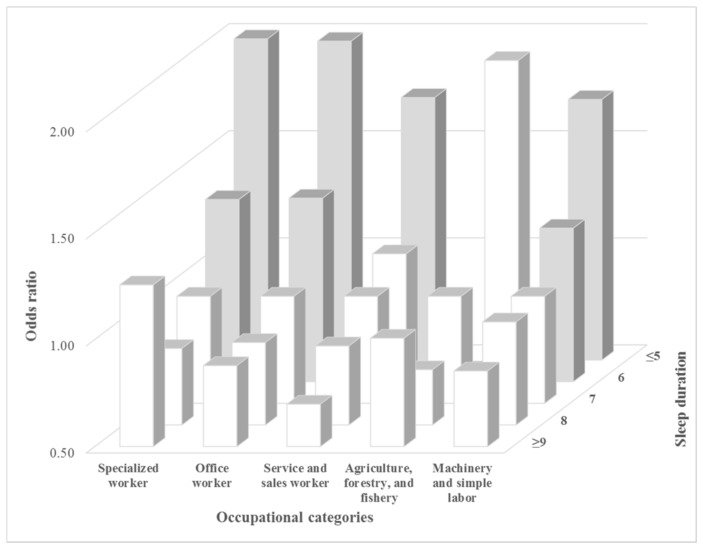
Subgroup analysis results for high-stress awareness according to occupational categories and sleep duration. Gray bar shows that the results of the multiple logistic regression were statistically significant (*p*-value < 0.05). Analyses were adjusted for the following covariates: age, educational level, household income, occupational categories, marital status, physical activity, alcohol consumption, smoking status, BMI, perceived health status, depressed mood, and chronic disease.

**Figure 2:**
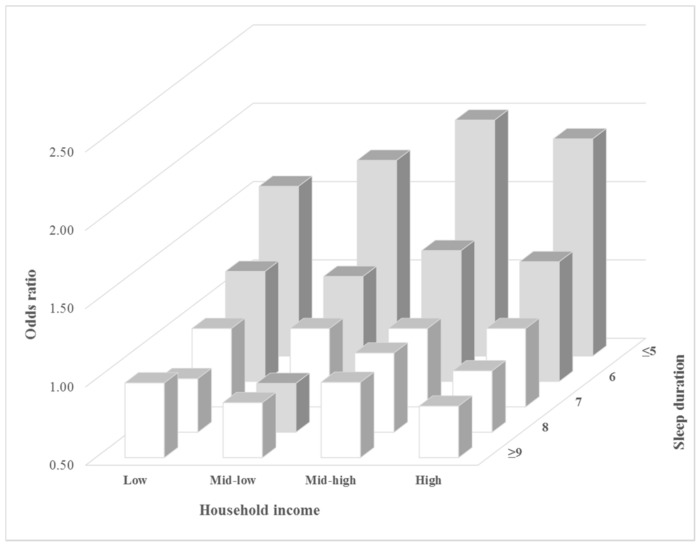
Subgroup analysis results for high-stress awareness according to household income and sleep duration. Gray bar shows that the results of the multiple logistic regression were statistically significant (*p*-value < 0.05). Analyses were adjusted for the following covariates: age, educational level, household income, occupational categories, marital status, physical activity, alcohol consumption, smoking status, BMI, perceived health status, depressed mood, and chronic disease.

**Figure 3:**
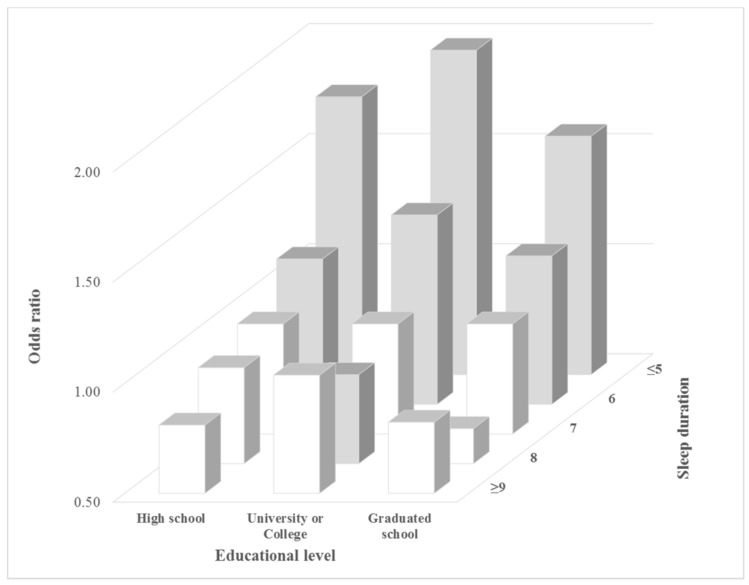
Subgroup analysis results for high-stress awareness according to educational level and sleep duration. Gray bar shows that the results of the multiple logistic regression were statistically significant (*p*-value < 0.05). Analyses were adjusted for the following covariates: age, educational level, household income, occupational categories, marital status, physical activity, alcohol consumption, smoking status, BMI, perceived health status, depressed mood, and chronic disease.

Figure 1, Figure 2 and Figure 3 shows the subgroup analysis comparing the odds ratios for high stress awareness for different factors according to sleep duration. In the occupational categories, those in specialized work had a high odds ratio for high stress awareness compared to those in other forms of work when they slept for five or less hours (OR: 2.03, 95% CI: 1.76–2.34). According to household income, the mid-high income group was a higher odds ratio for high stress awareness than others when they slept for <5 h compared to 7 h of sleep (OR: 2.01, 95% CI: 1.80–2.24). The low income group had the lowest odds ratio for high stress awareness when they slept for <5 h compared to others (OR: 1.58, 95% CI: 1.36–1.84). According to educational level, the university or college group’s odds ratio was 1.97 (95% CI: 1.79–2.18), the high school group was 1.76 (95% CI: 1.60–1.94), and graduate school was 1.58 (95% CI: 1.22–2.05).

## 4. Discussion

The main finding of this study is that short sleep duration might be associated with an increased odds ratio for perceived stress compared to adequate sleep duration. This result was shown in all occupational categories, except for agriculture, forestry and fishery. Particularly, the odds ratio for high stress awareness was higher for specialized workers, office workers and those with mid-high household income, than other factors. A previous study on the association between sleep duration and perceived stress showed that sleeping might be helpful in alleviating stress. Short duration of sleep increases blood pressure, increases evening cortisol and insulin levels, and is a chronic stressor [16,17]. It could also lead to several diseases such as type 2 diabetes mellitus, metabolic syndrome, depression, and hypertension [18,19,20,21,22].

According to age group, younger workers had high stress awareness compared to older workers. A previous study focusing on the stress of Korean workers showed same results that younger workers had higher perceived stress than old workers [23]. We may guess that the older workers are relatively stable, and the job stability and job compliance are higher than younger workers [24]. Compared to women workers, men workers had low perceived stress. Several studies found that women are more likely to engage in high strain work and that men have higher job control than do women [25,26,27]. Among health behavior factors, people who were smokers, drinkers, unhealthy, and depressed had high odds ratio compared to those who were not. Smokers might think that smoking would help to reduce stress. However, smokers had slightly high stress levels compared to non-smokers and smoking cessation leads to relieve perceived stress [28]. Previous studies also support that cigarette smoking was positively correlated with perceived stress [29,30,31]. Moreover, individuals who perceived poor health tended to get more stress than those who perceived good health [32]. The relationship between depressive mood and perceived stress was also revealed by previous study [33]. Therefore, we controlled those lifestyle factors which affect correlation for the association between sleep duration and perceived stress. However, these findings should be interpreted carefully because there may be a possibility of reverse causality.

Salaried workers with high educational level had high stress awareness than those who had low educational level. For household income, on the other hand, salaried workers who had low household income had high stress awareness. Low educational level and low household income have been known to be associated with stress. Previous studies showed that people who had low education levels had high levels of stress as they were likely to live under circumstances of higher population density, noise, discrimination, and poor access to healthcare [34,35,36]. In this study, however, the opposite was the case, with the low educational level group showing a lower odds ratio for high stress awareness compared to the other groups. Therefore, we performed a subgroup analysis for educational level according to sleep duration and there were reverse results, such that low education level had a lower odds ratio for high perceived stress compared to graduate school, university, or college workers when they slept for five or less hours. These results might indicate that high school or lower education level workers were likely to work on machinery and simple labor in South Korea, therefore, they might be getting less stressed compared to those with high educational level. A previous study showed that machinery and simple labor work were less stressful than other office work or service and sales work. Although they might work physically hard and get exhausted, they might work with less emotional and mental stress compared to other job categories and tend to close from work earlier than those engaged in specialized work and office work. In addition, it might be possible that they were trained in the laboratory of a graduate school. Graduate school students have to study a lot more than other students, and at the same time they have to do a lot of research and projects as well as work out a thesis. The previous study reported that university or college education level workers might be likely to have longer working hours and get highly stressed and not enough leisure time [37,38]. Moreover, over half of the population has graduated from university or college, and they usually engage in specialized work, office work, or service and sales work in South Korea [39].

South Koreans have the second-longest number of working hours in the world. South Korean salaried workers spend at work annually, which is 305 h more than the average figure among OECD member countries in 2015–2016. Consequently, office and specialized workers might have no time to rest enough and they spent almost time in their work place instead of the home. Those business circumstances have been induced until now and could cause social problems such as depression, chronic stress, and suicide [40,41,42,43,44]. These results may eventually lead to national problems as well as problems for individuals. Therefore, there is the need to change the working environment to improve the quality of workers’ lives at the national level. 

Our study has several limitations. First, it might not fully prove the causality between sleep duration and high stress awareness as this study is a cross-sectional study. Thus, the results should be interpreted carefully and further research needs to be uncovered the association between sleep and stress with a longitudinal view. Second, we could not consider the quality of sleep and the sleep duration by weekdays and weekends each because of limitations of the data. Third, we could not consider shift types—for example, nightshifts—because of data limitations. Fourth, the data were collected by a self-reported survey, therefore, there may be recall bias and under- or overestimation. Fifth, it may be possible that we did not fully include all variables affecting the results although we included several independent variables. Sixth, this study could not use the index of stress awareness as there were no questionnaires to measure the objective scoring index. Finally, we could not include working hours, therefore, there is the need for the further studies that consider working hours.

Despite these limitations, this study has several strengths. First, we used data from a nationwide survey; therefore, it would increase the representativeness of the data for the general South Korean population. Compared with previous studies, this study focuses on the sleep duration and stress awareness with occupational categories and socioeconomic status for only salaried workers. Therefore, it could serve as evidence for improving work circumstances in South Korea.

## 5. Conclusions

In summary, stress awareness is associated with short sleep duration; specialized workers, office workers, workers with household income above mid-high levels, and those who graduated from university or college especially need to sleep adequately in order to manage stress. In the long run, optimal work circumstances which observe weekly work hour limits or restrict frequent drinking subcultures in their workplace are much needed to improve the quality of life of salaried workers. Although it is not easy to change the work circumstance immediately in South Korea, we wish that this study could be the basis for changing company culture to guarantee workers’ health and quality of life. In addition, there is the need to further study the association between sleep duration and perceived stress considering working hours.

## Figures and Tables

**Table 1:** The general characteristics of the study population in this study.

Variables	Total (*N* = 67,425)	Stress Awareness				
		High (*N* = 19,207)		Low (*N* = 48,218)		*p*-Value
		*n*	%	*n*	%	
**Sleep duration**						<0.0001
≤5	10,158	3931	38.70	6227	61.30	
6	23,750	7143	30.08	16,607	69.92	
7	23,309	5754	24.69	17,555	75.31	
8	9140	2120	23.19	7020	76.81	
≥9	1068	259	24.25	809	75.75	
**Sex**						<0.0001
Men	35,844	10,104	28.19	25,740	71.81	
Women	31,581	9103	28.82	22,478	71.18	
**Age (years)**						<0.0001
20–29	8630	3214	37.24	5416	62.76	
30–39	16,361	5749	35.14	10,612	64.86	
40–49	19,197	5545	28.88	13,652	71.12	
50–59	15,323	3375	22.03	11,948	77.97	
≥60	7914	1324	16.73	6590	83.27	
**Educational level**						0.0004
Under high school	33,627	8379	24.92	25,248	75.08	
University or College	30,113	9715	32.26	20,398	67.74	
Graduated school	3685	1113	30.20	2572	69.80	
**Household income**						0.0063
Low	13,325	3640	27.32	9685	72.68	
Mid-low	13,910	3926	28.22	9984	71.78	
Mid-high	24,891	7140	28.69	17,751	71.31	
High	15,299	4501	29.42	10,798	70.58	
**Occupational categories**						<0.0001
Specialized worker	13,369	4184	31.30	9185	68.70	
Office worker	17,519	5704	32.56	11,815	67.44	
Service and sales worker	11,982	3580	29.88	8402	70.12	
Agriculture, forestry, and fishery	560	123	21.96	437	78.04	
Machine and simple labor	23,995	5616	23.40	18,379	76.60	
**Marital status**						0.0256
Married-cohabiting	46,732	12,538	26.83	34,194	73.17	
Married-divorce, bereavement, and separation	6392	1658	25.94	4734	74.06	
Single	14,301	5011	35.04	9290	64.96	
**Physical activity**						<0.0001
High	20,397	5965	29.24	14,432	70.76	
Moderate	23,243	6320	27.19	16,923	72.81	
Low	23,785	6922	29.10	16,863	70.90	
**Alcohol consumption**						<0.0001
Yes	45,446	13,710	30.17	31,736	69.83	
No	21,979	5497	25.01	16,482	74.99	
**Smoking status**						<0.0001
Current smoker	15,935	5655	35.49	10,280	64.51	
Ex-smoker	12,169	2903	23.86	9266	76.14	
Non-smoker	39,321	10,649	27.08	28,672	72.92	
**BMI**						0.0034
Normal and underweight (<23)	18,144	5518	30.41	12,626	69.59	
Overweight (23-25)	16,675	4403	26.40	12,272	73.60	
Obesity (≥25)	32,606	9286	28.48	23,320	71.52	
**Perceived health status**						<0.0001
Healthy	31,548	7,151	22.67	24,397	77.33	
Normal	30,652	9660	31.52	20,992	68.48	
Unhealthy	5225	2396	45.86	2829	54.14	
**Depressed mood**						<0.0001
Yes	3488	2183	62.59	1305	37.41	
no	63,937	17,024	26.63	46,913	73.37	
**Chronic disease**						<0.0001
Yes	4613	1913	41.47	2700	58.53	
No	62,812	17,294	27.53	45,518	72.47	

**Table 2:** Results for high-stress awareness according to factors.

Variables	High Stress Awareness	
	Adjusted OR	95% CI
**Sleep duration**		
≤5	1.86	(1.74–1.98)
6	1.27	(1.20–1.33)
7	1.00	
8	0.91	(0.84–0.98)
≥9	0.92	(0.76–1.10)
**Sex**		
Men	0.86	(0.81–0.92)
Women	1.00	
**Age (years)**		
20–29	3.44	(3.01–3.90)
30–39	2.93	(2.64–3.26)
40–49	2.16	(1.95–2.38)
50–59	1.43	(1.29–1.59)
≥60	1.00	
**Educational level**		
High school	0.83	(0.75–0.93)
University or College	0.93	(0.84–1.02)
Graduated school	1.00	
**Household income**		
Low	1.14	(1.05–1.23)
Mid-low	0.99	(0.93–1.06)
Mid-high	0.99	(0.94–1.05)
High	1.00	
**Occupational categories**		
Specialized worker	1.35	(1.25–1.45)
Office worker	1.43	(1.39–1.58)
Service and sales worker	1.32	(1.22–1.40)
Agriculture, forestry, and fishery	0.91	(0.66–1.26)
Machine and simple labor	1.00	
**Marital status**		
Married-cohabiting	1.10	(1.03–1.18)
Married-divorce, bereavement, and separation	1.07	(0.97–1.19)
Single	1.00	
**Physical activity**		
High	1.06	(1.00–1.12)
Moderate	0.93	(0.88–0.98)
Low	1.00	
**Alcohol consumption**		
Yes	1.20	(1.14–1.27)
No	1.00	
**Smoking status**		
Non-smoker	1.00	
Ex-smoker	1.10	(1.02–1.18)
Current smoker	1.64	(1.53–1.75)
**BMI**		
Normal and underweight (<23)	1.08	(1.02–1.14)
Overweight (23-25)	1.01	(0.95–1.07)
Obesity (≥25)	1.00	
**Perceived health status**		
Healthy	0.59	(0.56–0.62)
Normal	1.00	
Unhealthy	2.06	(1.90–2.24)
**Depressed mood**		
Yes	3.92	(3.57–4.31)
no	1.00	
**Chronic disease**		
Yes	1.44	(1.32–1.57)
No	1.00	
